# Exploiting the RSSI Long-Term Data of a WSN for the RF Channel Modeling in EPS Environments

**DOI:** 10.3390/s20113076

**Published:** 2020-05-29

**Authors:** Roddy A. R. Antayhua, Maicon D. Pereira, Nestor C. Fernandes, Fernando Rangel de Sousa

**Affiliations:** 1 Radio Frequency Laboratory at the Department of Electrical and Electronics Engineering, Federal University of Santa Catarina, Florianopolis, SC 88040-900, Brazil; roddy.romero@ifsc.edu.br (R.A.R.A.); maicon.deivid@ufba.br (M.D.P.); 2 Department of Electrical Engineering, Federal Institute of Santa Catarina, Itajai, SC 88007-303, Brazil; 3 Department of Electrical and Computer Engineering, Federal University of Bahia, Salvador, BA 40210-630, Brazil; 4 Traceback Technologies, Florianópolis, SC 88090-145, Brazil; nestor@traceback.com.br

**Keywords:** IIoT, WSN, RSSI, power plants, RF channel model, EMI

## Abstract

In this paper, we propose a methodology to use the received signal strength indicator (RSSI) available by the protocol stack of an installed Wireless Sensor Network (WSN) at an electric-power-system environment (EPS) as a tool for obtaining the characteristic of its communication channel. Thereby, it is possible to optimize the settings and configuration of the network after its deployment, which is usually run empirically without any previous knowledge of the channel. A study case of a hydroelectric power plant is presented, where measurements recorded over a two-month period were analyzed and treated to obtain the large-scale characteristics of the radiofrequency channel at 2.4 GHz. In addition, we showed that instantaneous RSSI data can also be used to detect specific issues in the network, such as repetitive patterns in the transmitted power level of the nodes, and information about its environment, such as the presence of external sources of electromagnetic interference. As a result, we demonstrate the practical use of the RSSI long-term data generated by the WSN for its own performance optimization and the detection of particular events in an EPS or any similar industrial environment.

## 1. Introduction

We are currently living in the era of Industrial Internet of Things (IIoT), in which several disruptive technologies have converged to change the way modern companies manage their manufacturing and industrial processes [1]. For example, the energy sector is embracing this new technological trend aiming at increasing production efficiency, reducing emissions, and improving employee safety and supply chain traceability [2,3,4]. In this context, Wireless Sensor Networks (WSN) have been progressively used in industrial environments as a result of the IIoT trend, which has helped to bring down old paradigms in which wireless communication was perceived as less reliable and unsafe [5,6,7,8,9,10]. For instance, WSNs have already been explored in power plants for different applications [11,12,13,14].

A WSN deployed in an industrial environment must assure an acceptable degree of reliability and security, thus, robust network design is required. This means that it is important to acquire a minimum knowledge of the communication channel, which certainly entails a characterization task. Traditional methods, in which the propagation channel is evaluated through point-to-point communication links prior to the network installation, are frequently not executed in complex Electric Power System (EPS) environments since it usually demands specific laboratory equipment and, more critically, the measurements are performed non-simultaneously over the expected communication area in the EPS facility and over limited time periods for each point, given the hardware and the several measurement steps required [15,16]. Wireless modules working in pairs have also been used for characterization purposes in power generation and distribution environments; however, this method presents the same related issues of limited time periods and measurement data [5,17].

Similar to other EPS environments, power plants are complex facilities that present strict regulations for safety, making the wireless channel analysis difficult with the conventional point-to-point methods. Therefore, it is often the case that the radio nodes of the WSN are initially located near the spots where specific equipment needs to be monitored, and then the number of routers and their hardware settings are adjusted following a trial-and-error procedure until a stable topology is achieved. This recurrent procedure may not yield an optimum result from the standpoint of the number of nodes, redundancy, power consumption, etc.

Only a few communication channel studies have been conducted aiming at the specific scenario of power plants using traditional methods [11,17,18]. In Reference [11], measurements were conducted in an indoor environment at a nuclear power plant for path-loss and fading characterization. These tests were focused on IEEE 802.11 compliant equipment and were performed with a radiofrequency (RF) transmitter and a spectrum analyzer operating at the 910 MHz and 2.4 GHz ISM frequency bands. Only the path loss coefficient obtained from measurements was reported by the authors: n=1.86. The study reported in Reference [18] presented measurement results on the noise and interference levels in the communication channel between 500 MHz and 5.8 GHz for the purpose of verifying the WSN performance on a fossil fuel power plant. A broadband signal recorder-and-generator was used to record the signals in the time domain and a spectrum analyzer for the information in the frequency domain. The authors did not present the path-loss coefficient or shadowing results since they focused on the identification of noise or interference in the channel. No communication signals were detected on the ISM bands, but the results showed that the noise floor ranged from −79 dBm to −85 dBm and an intermittent interference was detected that increased the noise floor by 43 dBm in all bands. The interference source was not identified. In Reference [17], channel measurements and channel modeling for a medium-scale coal-fired power plant with a maximum power output of 175 MW were presented. For the test procedure, wireless modules operating in the 2.4 GHz band complying with the IEEE 802.15.4 standard were used. These modules were systematically changed in position after registering the Received Signal Strength Indicator (RSSI) and Packet Error Rate (PER), which were then used to estimate the channel characteristics. Unique path-loss, shadowing, and fading characteristics compared to other indoor scenarios were found. Path-loss coefficients estimated were n=1.51 and 3.66, for line-of-sight (LOS) and non-LOS (NLOS) environments, respectively. The shadowing related lead to a standard deviation of 5.45 dB, following a log-normal distribution. The RSSI and PER measurements also showed that noise related to the operation of the power plant equipment did not significantly affect the quality of the communication.

As an alternative to the cited studies in which the propagation channel in a power plant was characterized through conventional methods, the authors have proposed in Reference [19] to use the RSSI data reported by the protocol stack level of the nodes from a deployed WSN for the channel characterization. In this way, the inherent information from the network can be used to understand the instantaneous and average large-scale characteristics of the communication channel and its change over time. The use of the RSSI data registered by the WSN nodes can then be used as a characterization method, and consequently, as a tool for improving the settings of the installed network.

The authors presented in Reference [19] the path-loss coefficient and shadowing deviation results extracted from the measurements registered in a power plant during a few days. In this paper, we present new results from data recorded over a two-month period. Through the analysis of this higher amount of data, long-term mean values are obtained and used as a basis for comparing the channel behavior to events related to operations on the power plant that occurred on particular days. Complementary to this, we discuss how the recorded data can reveal issues about the functioning of specific nodes. For example, through a cross-correlation analysis of the measured RSSI data of each bidirectional link, it is possible to estimate the rate of increase on the transmission power of each node, which is directly related to the quality of the links. Finally, we also demonstrate that the high-level RSSI values registered during recordings, which are treated as undesired information when modeling the channel, can be co-related to the electromagnetic interference (EMI) sources present in the power plant. Therefore, the possibility to use the WSN as a tool for detection and understanding these sources is raised.

As a result, the main contribution of this work is the proposal of a methodology to obtain the long-term channel propagation characteristics using the RSSI data reported in a deployed WSN. This method may be a way of overcoming the lack of measurements campaigns before the WSN installation in EPS environments or other similar industrial environments involving large complex facilities and rigid safety regulations in which traditional point-to-point methods are difficult to perform. Through the knowledge of the channel, improvements in the network can be planned and executed in the medium-to-long term, such as redefining the default transmission power level of the nodes or adding extra routing nodes. Complementary to this, this work also shows additional useful information that can be extracted from the long-term registered data regarding the behavior of the network, hardware issues in the nodes, and EMI sources.

The rest of the paper is organized as follows. The network deployment, measurement environment, and the explanation of the channel characterization methodology is presented in Section 2. A discussion about the network behavior from a qualitative analysis of the RSSI data is done in Section 3. Moreover, the results obtained from the power plant measurement during a two-month period following the procedure described in Section 2 is presented. In Section 4, two complementary examples of the use of the RSSI data regarding the nodes transmission power and the EMI sources are presented and discussed. Finally, the conclusions and future work are left for Section 5.

## 2. Measurement Environment and Methodology

### 2.1. Measurement Setup and WSN Deployment

**Figure 1:**
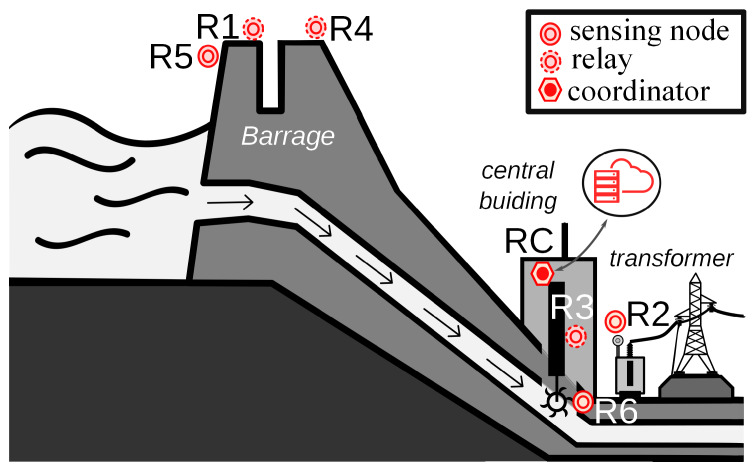
Wireless Sensor Network (WSN) installed in a hydroelectric power plant.

For this work, we were provided with data from a WSN already installed at the hydroelectric plant *Cachoeira Dourada* (MG, Brazil). The position of the node radios in the power plant is sketched in Figure 1. In addition, a description of their roles and location is given in Table 1. Only three of the radios were used to gather information from sensors, the others were used for network routing. The radios were deployed in a 92 m × 78 m area and distributed among three main locations: the barrage, the central building, and where the step-up transformers and high-voltage cables are present. Radios R1, R4, and R5 were placed at the top of the barrage, which is about 22 m high. Radios RC, R3, and R6 were installed inside the central building, which is about 12 m high, being radios R3 and R4 about 20 and 30 m below RC, respectively. Finally, radio R2 was installed next to one of the transformers, about 4 m above ground level. The exact distance between the radios is specified in Table 2. Due to the heterogeneous environment of the facility, both LOS and NLOS conditions existed between the radios.

The radios operated in the 2.4 GHz band under the Zigbee 3.0 protocol and in a mesh configuration. The theoretical receiver sensitivity of each node was −95 dBm. In addition, the default transmitter output power level reaching the radio omnidirectional (monopole) antenna was set to 11 dBm; however, this power could automatically increase at discrete-value power steps if link quality needed to be guaranteed or improved. Either batteries supported by solar panels or the mains were used as power supplies. More details on the radio modules hardware can be found in Reference [19]. The nodes were configured to operate only in the Zigbee channel 15 (2425 MHz). All the radios were set as routers (relays), so each one was able to establish different routes to communicate up to the coordinator. The coordinator uploaded the radios data to a local database.

### 2.2. Channel Characterization Methodology

**Figure 2:**
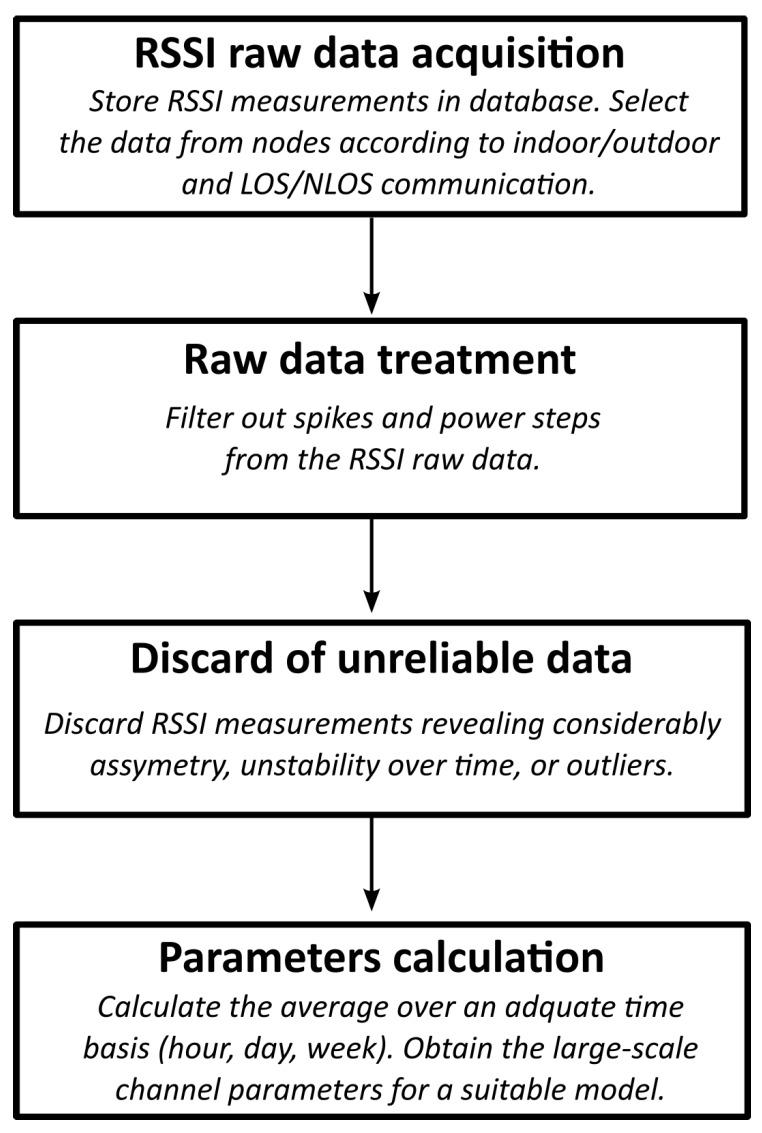
Channel modeling methodology. RSSI = received signal strength indicator; LOS = line-of-sight; NLOS = non-line-of-sight.

The procedure to extract the channel parameters from the recorded data can be divided into four steps, as depicted in the flux diagram in Figure 2. First, a pre-selection of the nodes is necessary based on the knowledge of their locations. As the registered RSSI raw data from all the radios is available at the local database, only the nodes that are suitable for the channel characterization according to the type of communication link (indoor/outdoor and LOS/NLOS) need to be taken into account.

After this, the collected raw data should be treated before the calculation of the large-scale parameters of the communication channel. A first issue to be addressed is related to the noisy environment regarding industrial locations, such as power plants. Due to the presence of high-power equipment, EMI may be generated which can lead to misreadings in the RSSI values, generally registered as abrupt peaks resembling spikes. Those spikes should be filtered out as they are not related to changes in the communication channel. A second issue is related to the transmission power of each node. Since the radios are able to automatically increase their transmission power to improve their link quality, these increments are registered by other radios as transitory pulses in the RSSI. These pulses need to be removed, otherwise, this could lead to miscalculation of the path-loss coefficient.

The next step, previous to the channel parameters calculation, concerns the discard of unreliable data. This is necessary due to eventual hardware issues or non-stable communication between nodes. For example, an excessive asymmetry level can be noticed in any of the radios registered RSSI data, indicating an issue on what should have been a bidirectional symmetric link. Naturally, if a calibration procedure of the WSN radios is done previous to its installation, the asymmetry information may be corrected in this step. In addition to this, unstable connections are noticed when the corresponding RSSI registered data is very low or even sporadic compared to the rest of the stable links. Based on the RSSI registered values by each radio pairs, and on the periodicity of these data, some measurements can be considered as outliers, and the information of the corresponding nodes should be discarded.

Finally, the data can be used for the extraction of the parameters of the channel model. First, the instantaneous RSSI data for each node, which was measured and recorded at specific time intervals, is averaged over convenient measurement periods according to the time window for which the parameters are to be used. For example, an hour basis average may be useful to keep a record of the channel behavior during a day. After this, these values are used to obtain the large-scale communication channel parameters, which includes the path-loss coefficient and the shadowing deviation. The choice of the channel model may be any that better suits the industrial environment. For the purpose of this work, a single-slope log-distance model was used due to its simplicity and versatility [19,20]. This model and the procedure for obtaining its parameters are briefly described next.

### 2.3. Large-Scale Channel Parameters

The path loss (PL), which estimates the power attenuation with the distance (*d*), is described by

(1) $$PL\left[\mathrm{dB}\right]=PL\left[d_{0}\right]+10n\log\left(d/d_{0}\right),$$

where *n* is the path-loss coefficient, which depends on the propagation environment, and $d_{0}$ is a reference distance close to the transmitter. The path-loss measurement ($PL_{m}$) was calculated from the difference between the known transmitted power at each radio and the averaged RSSI, as

(2) $$PL_{m}\left(d\right)\left[\mathrm{dB}\right]=P_{tx}-RSSI.$$

A fitting curve was then obtained from the measured path-loss data to identify the path-loss coefficient (*n*) in Equation (1). The fitting was done using a linear least-squares regression to find the coefficients, $P_{1}$ and $P_{2}$, that fit the data to $PL_{fit}\left(d\right)$, using

(3) $$PL_{fit}\left(d\right)=P_{1}\log\left(d\right)+P_{2}.$$

In addition to the path-loss coefficient, shadowing deviation was also calculated from the measurement data. Shadowing accounts for the difference in path-loss values between two objects in different locations and at the same distance to a common transmitter. This difference arises due to the presence of obstacles along each of its corresponding propagation paths. Commonly, the shadowing is modeled by a random variable with Gaussian distribution with a zero mean and a standard deviation obtained from the shadowing samples (*X*). Since the random deviation of the measured path-loss, $PL_{m}$, occurs around a mean value and the fitted path-loss, $PL_{fit}$, is essentially the mean path-loss over distance, the shadowing samples can be calculated using [21]

(4) $$X=PL_{m}\left(d\right)-PL_{fit}\left(d\right).$$

Finally, normality tests were applied to these results to verify the statistical distribution of the samples [22].

## 3. Analysis and Results from the Collected Data

### 3.1. Qualitative Analysis of the Network Behavior

**Figure 3:**
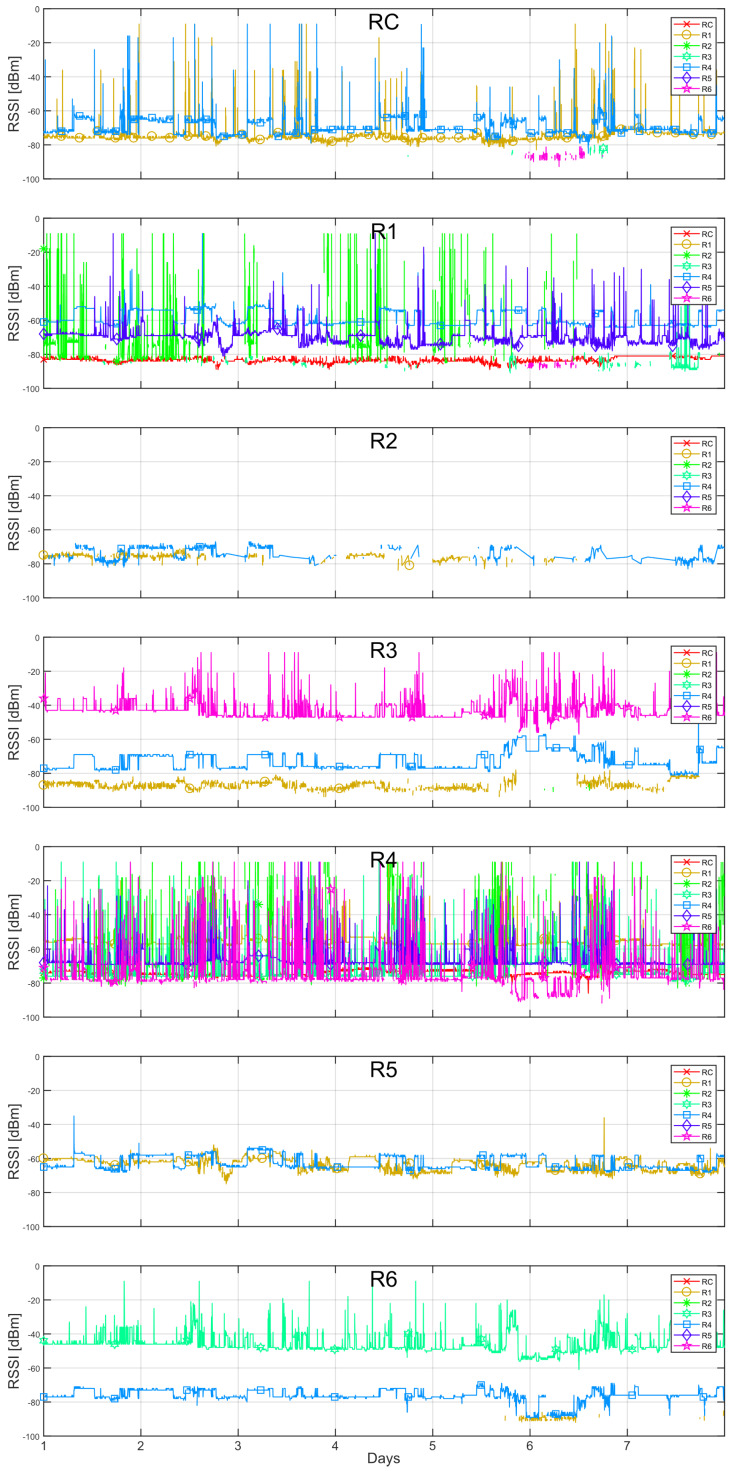
Instantaneous RSSI from the neighbors reported by each radio in a week period.

The instantaneous RSSI data recorded from each of the radios is plotted as a function of time in Figure 3. For each graph in this figure, the nodes listed in the legend to the right correspond to the transmitters and the name at the top center of each graph corresponds to the receiver node. This data corresponds to one week, which was particularly chosen as an illustrative example due to some specific events that occurred during that period. In general, we can observe spikes in the RSSI values in almost all radios. As briefly discussed in Reference [19], an RSSI of −9 dBm corresponds to the maximum value of power that can be registered by node radios. In this case, they also represented an abandoned communication between two neighbor radios. Furthermore, the spikes below −9 dBm were attributed to EMI, which is further discussed in Section 4.

**Figure 4:**
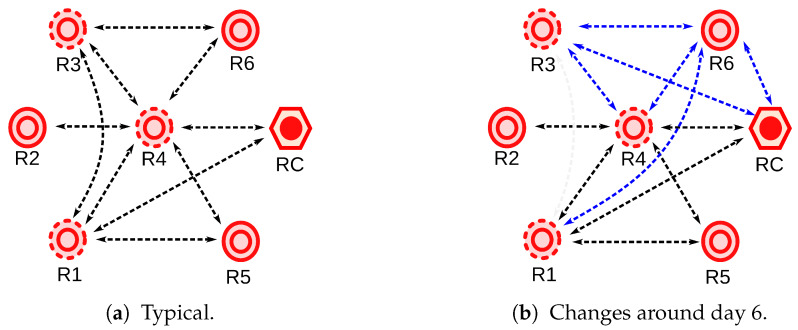
The topology of the mesh network extracted from RSSI measurements shown in Figure 3.

It can be seen that the coordinator radio RC perceives only two radios, R1 and R4, with some spikes in both cases. Radio R1 perceives radios R2, R3, R4, and R5, where the first is reported mostly with spikes. Some spikes were also reported for the remaining radios, however, in less amount. Radio R2 perceives only R1 and R4, however, intermittently, and radio R3 perceives radios R1, R4, and R6, with spikes only present in the latter. Radio R4 perceives all radios in the network, and registers several spikes from them, mostly from R2. A closer inspection revealed that a stable RSSI was only reported from the coordinator radio. Radio R5 perceives radios R1 and R4. Finally, R6 perceives radios R3 and R4, with spikes showing in the former. A representative diagram of the network topology based on a usual mesh behavior based on the RSSI measurements is shown in Figure 4a. Here, the fact that radio R4 is a critical router through which the other radios reach the coordinator is better observed.

From the instantaneous RSSI, it can also be noticed, that around day no. 6, there was an event which caused considerable variations on the recorded RSSI in comparison to the rest of the days. For example, it made the coordinator radio to perceive in some moments radios R3 and R6. The event also caused radio R1 to perceive radio R6, and to stop noticing radio R3. In addition, it made radio R3 to perceive a higher RSSI from radio R4, to perceive radio R6 with many spikes and to lose detection of radio R1. Moreover, radio R4 perceived radios R3 and R6 with higher and lower intensity, respectively, but the rest remained normal. Radio R6 perceived radios R3 and R4 with lower intensities and it started perceiving radio R1. The only radio that did not record any particular variation was radio R5. These changes are represented in Figure 4b.

After analyzing the observed changes in RSSI recordings of radios R1, R3, R4, and R6 during this day (i.e., higher RSSI between radios R3 and R6 and lower RSSI between radios R3 and R4, R3, and R1, and R4 and R6), it could be inferred that there was a change in the communication channel surrounding radios R3 and R6. This could have been caused by a particular change on the physical setting inside or close to the central building over the referred period. For instance, we knew that there is a large moving crane inside the main building of the power plant which is used to move machinery, including the power plant turbines, for maintenance. However, we did not have access to the chronology of these events to associate it with the observed events.

**Figure 5:**
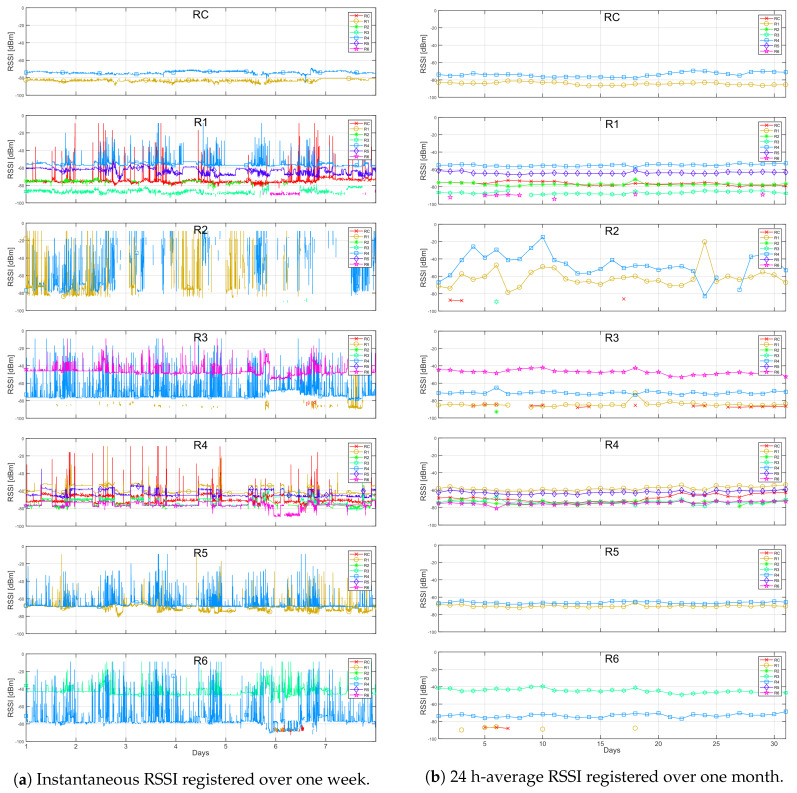
RSSI data corresponding to each radio as seen by its neighbors.

Additional facts about the network behavior can be better visualized by plotting the reciprocal RSSI measurement of a radio, that is, how a particular radio is perceived by its neighbors. This is shown in Figure 5a. In this figure, the nodes listed in the legend correspond to the receivers and the name at the top center of each graph refers to the transmitter. By comparing this to Figure 3, it is easier to observe the symmetry of the RSSI level recorded by each pair of radios. Regarding this symmetry, some offsets in all radios were observed. This was expected since the absolute values of the RSSI registered at each radio module were not calibrated to correct for intrinsic errors related to the received power meter circuitry.

Particularly, radio R1 presented the largest offsets among all radios, registering a value 4 dB lower, in average, than the perceived by its neighbors.

In the same plots, temporary power increments that appeared as pulses in the RSSI can be observed, for example, from the neighbors of radio R4. These pulses represented a fixed increment on the transmission power level of the nodes during a certain period of time. They did not correspond to a change of the communication channel as the changes in the RSSI level were perceived by all of the neighbors, indeed. A closer inspection in the RSSI data recorded by the rest of the radios revealed the presence of similar power pulses, over shorter periods though.

A visualization of the averaged RSSI data over time also helped in the analysis regarding the unstable links and some of the events registered on specific days. Figure 5b shows the 24-hour average RSSI reciprocal values over a month period. Almost all radios presented a relatively stable value over this period, except for measurements from radio R2, in which the amount of valid registered data was very low compared to the rest of the radios.

### 3.2. Estimation of the Large-Scale Parameters of the Communication Channel

#### 3.2.1. Raw Data Treatment and Nodes Selection

In this work, only NLOS indoor-outdoor data was used for the characterization of the channel, since they were more significant in number and future expansions of the network would likely include more radios working in this condition. The RSSI spikes in the recordings were filtered out; that is, all values above a specific threshold (15 dB above the RSSI mean of that day) were discarded since they were not attributed to changes in the channel as explained in Section 2.

Some outliers were also evidenced from measurements. For example, the RSSI values registered between radios R2 and R3, which were only recorded a very few times during one day in the first week. Therefore, they were excluded to prevent altering the results. This issue could be critical if a small number of available radios in the network is deployed, as was in the case for the studied power plant. In this situation, considering radios R2 and R3 could lead to an error of more than 100% in the path-loss coefficient value. Finally, as evidenced in the instantaneous RSSI plot, radio R1 presented considerably asymmetry compared to the rest of the radios, so its corresponding measurements were discarded.

After treating the data and removing the outliers, the average of the RSSI data was taken on a 24-h basis.

#### 3.2.2. Path Loss and Shadowing Results

**Figure 6:**
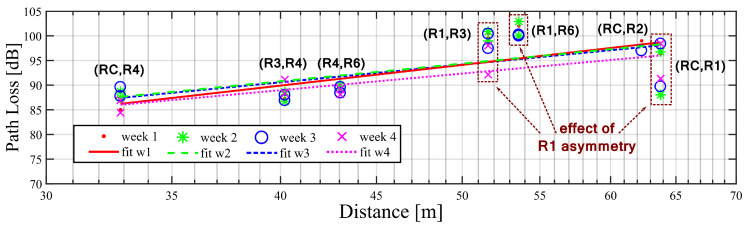
Path loss measurements points over distance of the radio pairs and linear regression fittings during different weeks over the first month of measurements.

The path loss between nodes, the path-loss coefficient, and shadowing factor were calculated following the procedure described in Section 2.3. Figure 6 shows the path loss measurements and the best linear regression based in these measurements for a period of one month divided in weeks. The measurements data for each week were obtained from the mean result of daily averages. Every measurement point in the plot is identified with its corresponding node name and, for each distance, there are at least two nodes data per period representing a reciprocal radio pair measurement. The residual difference between them are attributed to the RSSI calibration offsets and to some amount of error during the process of removal of the RSSI spikes and pulses. In this figure, the measurements regarding radio R1 and its pairs were included to show the high asymmetry level mentioned earlier. We decided to discard the RSSI recordings from R1 (but not its reciprocal) for the channel characterization.

**Figure 7:**
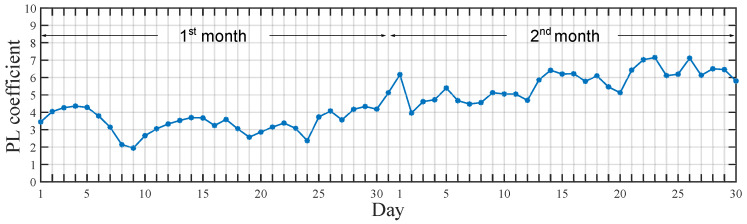
Daily variation of the path-loss coefficient (*n*) over two months of measurements.

From the linear regression fitting of the measured data, the path-loss coefficient and shadowing deviation were estimated. The path-loss coefficient per-day evolution over a two-month period is shown in Figure 7. A rising trend in the path-loss coefficient was observed for the second month. However, the correlation of this increase with the events in the power plant cannot be confirmed due to non-accessible sensible/confidential information regarding the operation of the power plant, like the activation of specific machinery, or any spatial change nearby the radios, like the movement of vehicles or bulky machinery, or either the weather conditions over those days.

A summary of the results is presented in Table 3. The mean value of the path-loss coefficient during the two-month period resulted in n≈4.6. Deviations from this value showed in the daily variation of the path loss in Figure 7 reveals the impact of environmental conditions and power-plant dynamics over time.

## 4. Additional Uses of the Long-Term RSSI Data

Besides the propagation channel obtained by using the averaged value of the pre-processed RSSI data over time, some other useful information can be extracted from monitoring the changes of the RSSI values over time. For example, since a big amount of data is collected, mean values of the channel parameters that characterize the default conditions of the plant can be defined and, eventually, gradually updated, so deviations from these values could be continuously monitored.

Furthermore, the RSSI instantaneous data can also provide practical information from the network and the measurement environment. Two examples are shown next, one regarding the estimation of the network link efficiency from the rate of change of the transmitted power level of the radios, and the other concerning the detection of sources of EMI.

### 4.1. Detection of Transitory Variations Rate in the Transmission Power Level of the Radios

Since there is a relation between the rise in the transmission power level—characterized by the occurrence of pulses in the measured RSSI—and the link quality, the rate of occurrence of these pulses can serve as a metric for identifying possible improvements on the network configuration. For example, the fact that radio R4 is a critical router in the network - requiring it to always communicate successfully with the coordinator—could be related to the large time periods in which its transmission power level was raised, as discussed in Section 3.

Through correlation analysis, the rate of occurrence of pulses can be inferred, and, if this rate is above an specific threshold, it can be an indicator of a repeated difficulty for a radio node to establish a connection up to the coordinator. This diagnostic can be used to decide if, for example, an extra node is required in the network, or if the default transmitted power of a particular node should be increased.

To obtain the rate of occurrence of pulses, we performed the cross-correlation between the RSSI raw data recordings (only spikes filtered) of what radio *RX* sees of radio *RY* and vice-versa. The obtained cross-correlation coefficient (CC) at zero lag was low if a radio was constantly changing its transmission power level. When this coefficient was high, the opposite behavior could be inferred for the transmission power level.

The averaged zero-lag cross-correlation coefficients for each radio RX and its neighbors were obtained from the following procedure: Spikes from the RSSI raw data of each radio over a month period were removed. Then, this data was interpolated with one-minute steps, resulting in N-length vector of RSSI data for each radio. After this, the data was normalized by its average value (rssi). Next, the zero-lag cross-correlation coefficient for each radio pair, RX-RY, was calculated from:

(5) $$CC_{x,y}=\sum_{i=1}^{N}rssi_{x}\left(i\right)rssi_{y}\left(i\right).$$

Finally, the averaged results for each radio was obtained from:

(6) $$\bar{CC_{x}}=\frac{\sum_{i=1}^{n_{x}}CC_{x,i}}{n_{x}},$$

where $n_{x}$ is the number of the detected neighbors of radio RX.

The resulting $\bar{CC_{x}}$ for each radio is presented in Table 4. As noticed, all radios presented a relatively low value except the coordinator. This was expected since it was observed in Figure 3 and Figure 5a that those radios changed the transmitting power over time. Radio R4, which was one of the radios which varied its transmitting power during the largest periods, obtained an average CC of 0.3, which can be considered low for this test in comparison to 0.74 obtained for the coordinator. To determine if the rate of transitory pulses is significant, an adequate threshold value can be chosen.

### 4.2. EMI Detection

From the examination of the recorded RSSI of each radio done in Section 3, it was already highlighted the fact that many of the recordings presented sudden changes that resembled spikes in the received power. It was raised the hypothesis that there was a connection between the spikes and some source of EMI.

To support this hypothesis, we performed tests to a network installed in an indoor non-industrial environment [19]. In these tests, a software-defined radio platform was used to generate high-power radiofrequency continuous wave pulses in the network environment acting as radiofrequency blockers. From the RSSI recorded in some of the radios, we observed similar spikes than those reported in the power plant. We then reinforced the idea that those spikes could be related to some source of EMI with enough spectral energy within the working frequency band of the network. We also verified that those blockers would only make communication between radios to drop if they were enabled for a long time (>10 s).

To identify the EMI connection with the RSSI spikes reported by the nodes installed in the power plant, a secondary information source was used. It was known that possible sources of EMI at the power plant were the step-up transformers and the high-voltage cables that emerge from the transformers installed at each of the turbines [23]. These transformers were located at one side of the central building, opposite to the barrage, where the radio R2 was installed. As described in Table 1, this radio included a temperature sensor that monitored the oil reservoir of one of the transformers in the plant. When the transformer is turned on, the oil temperature rises. Thus, the temperature registered by radio R2’s temperature sensor is an indirect indicator of when this transformer was operative.

**Figure 8:**
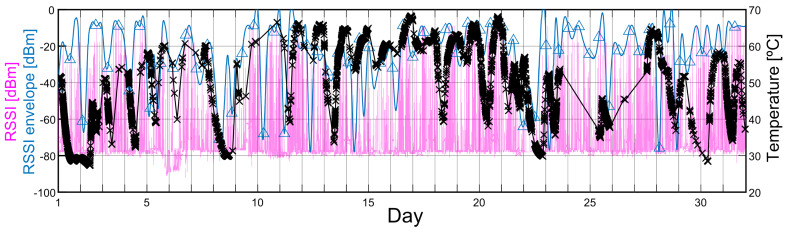
Curves showing the relation between the temperature response of one of the transformers (black line for interpolation and crosses for measurements) in the plant registered by radio R2 and the envelope (blue line) of the instantaneous RSSI of radio R6 registered by radio R4 (pink line).

Recalling the instantaneous RSSI shown in Figure 3, radio R4 recorded predominantly spikes, except from the coordinator. We hypothesized that the spikes were direct related to the EM radiation from transformers and its high-voltage cables. To confirm this, we plotted the RSSI recorded by radio R4 from each of its neighbors that presented spikes together with the temperature curve from the sensor connected to radio R2. An example of this is shown in Figure 8 for the R4 neighbor radio R6 during one of the recorded months. The temperature curve shows explicitly the temperature measurement recordings including a linear interpolation. The periods in which no temperature measurements were recorded are attributed to failures in the radio hardware due to battery issues. Nonetheless, a correspondence between the periods in which the transformer was active and the registered RSSI spikes are observed. Similar behavior was observed for the rest of the R4 neighbor radios.

This relation was further studied by cross-correlating the transformer temperature response with the envelope of the RSSI data including the recorded spikes. An example of the RSSI envelope obtained for radio R6 was included in Figure 8. The envelope was extracted by using a spline interpolation over a local maxima separated by a window size with a specific minimum number of samples. A similar procedure to the one described in Section 4.1 was performed to calculate the CC between the RSSI envelope and the transformer temperature response, although, this time, for several time-lag values. In all cases, a CC maximum value was observed at around a 10-minute lag approximately, which means that the temperature curve is delayed from the RSSI envelope. This delay value was expected due to the transformer oil reservoir thermal inertia. Similar results were obtained from the other neighbors of radio R4 except for the coordinator, which did not present spikes.

These results indicated that there might be a measurable relationship between the RSSI collected by the radios and the interference from high-voltage sources. Fortunately, these interferences did not show evidence of being capable of dropping a communication link between the radios, which was in accordance with the tests performed at the WSN installed at the indoor non-industrial environment, and was also expected from the results presented in related studies [11,17]. Since the radios were able to register these events through the RSSI spikes, we raise the possibility of using the RSSI information as a technique for detecting EMI. In other words, the WSN could serve as an EMI sensor. If the data from all radios is used, it may also be possible to geolocalize the sources of interference [24,25]. This could be useful in industrial scenarios in which there is no previous knowledge of the sources of interference.

## 5. Conclusions and Future Work

Knowing the recent adoption of emerging technologies by the industrial sector, this work focused on the specific issue of the deployment of WSNs in electrical-power-system environments. It is often the case that the nodes of the network are installed and their settings, such as the number of nodes and location, are optimized empirically. This common procedure is preferred since a proper characterization of the propagation channel usually demands a considerable measurement effort, hindered by the complex structure and the strict safety regulations of power plants. Based on this, we have proposed a methodology to use the network as a tool for characterizing the propagation channel by using the available data of the protocol stack. We presented the study case of a hydroelectric power plant, in which the RSSI recorded by all the nodes of the network was used to obtain the mean path-loss coefficient and the shadowing statistics during a two-month period. A careful treatment of the data was performed after the visual inspection of time-domain RSSI information and the understanding of the mesh network behavior. As a result, we were able to record and analyze the evolution of the channel characteristics over this period and to detect possible issues in the network. The channel parameters and other information obtained can be used for the optimization of the WSN or a future expansion of the network deployed. In addition to this, some examples of the correlation results of the instantaneous recorded data were presented. This included the detection of some critical nodes where transmitted power was momentarily increased to ensure a reliable communication link. Moreover, it was shown that the spikes observed in the RSSI data presented a correlation with the operating regime of a step-up transformer. Thus, we also raised the possibility of using the WSN as a tool for EMI detection. In future developments, the results of the communication channel characteristics can be cross-correlated with other information of the power plant, such as the generated power rate or with the weather conditions. It is also worth noting that the proposed method can be applied to other channel propagation models than the single-slope log-distance model used in this work. Finally, automation of the data processing and big data techniques can be applied to manage larger quantities of information from the network considering, for example, a bigger WSN and recordings from larger periods of time.

## Figures and Tables

**Table 1:** Description of the nodes in the WSN.

Radio	Function	Location
RC	Coordinator	Office room at the central building
R1	Relay	Barrage
R2	Transformer temperature monitor	Transformer
R3	Relay	Central building
R4	Relay	Barrage
R5	Barrage water-level monitor	Barrage
R6	Well water-level monitor	Central building

**Table 2:** Distance (in meters) between the radios installed in the power plant.

	RC	R1	R2	R3	R4	R5	R6
RC	-	63.8	62.3	28.9	32.9	104	29.6
R1	63.8	-	48.7	51.6	33.7	44.1	53.6
R2	62.3	48.7	-	33.5	58.9	77.9	35.7
R3	28.9	51.6	33.5	-	40.2	90.1	5.1
R4	32.9	33.7	58.9	40.2	-	74.9	43.1
R5	104	44.1	77.9	90.1	74.9	-	90.8
R6	29.6	53.6	35.7	5.1	43.1	90.8	-

**Table 3:** Path loss and shadowing deviation results obtained from the RSSI data.

	Month 1				Month 2			
	Week 1	Week 2	Week 3	Week 4	Week 1	Week 2	Week 3	Week 4
*n*	4.06	3.09	3.11	2.91	5.1	5.9	6.03	7.03
σ [dB]	4.79	5.28	4.22	2.92	4.33	3.85	4.56	5.2

**Table 4:** Averaged cross-correlation coefficients obtained for all radios, as described by Equation (6).

Radios	RC	R1	R2	R3	R4	R5	R6
$\bar{CC}$	0.74	0.4	0.24	0.25	0.3	0.09	0.43
